# Dynamic Buffer Capacity in Acid–Base Systems

**DOI:** 10.1007/s10953-015-0342-0

**Published:** 2015-06-16

**Authors:** Anna M. Michałowska-Kaczmarczyk, Tadeusz Michałowski

**Affiliations:** Department of Oncology, The University Hospital in Cracow, Cracow, Poland; Faculty of Chemical Engineering and Technology, Cracow University of Technology, Warszawska 24, 31-155 Cracow, Poland

**Keywords:** Acid–base equilibria, Buffer capacity, Titration

## Abstract

The generalized concept of ‘dynamic’ buffer capacity *β*_*V*_ is related to electrolytic systems of different complexity where acid–base equilibria are involved. The resulting formulas are presented in a uniform and consistent form. The detailed calculations are related to two Britton–Robinson buffers, taken as examples.

## Introduction

Buffer solutions are commonly applied in many branches of classical and instrumental analyses [1, 2], e.g. in capillary electrophoresis, CE [3–5], and polarography [6]. The effectiveness of a buffering action at a given pH is governed mainly by its buffer capacity (*β*), defined primarily by Van Slyke [7]. The *β*-concept refers usually to electrolytic systems where only one proton/acceptor pair exists. A more general (and elegant) formula for *β* was provided by Hesse and Olin [8] for the system containing a *n*–protic weak acid H_*n*_L together with strong acid, HB, and strong base, MOH; it was an extension of the *β*-concept from [9]. The formula for *β* found in the literature is usually referred to the ‘static’ case, based on an assumption that total concentration of the species forming a buffering system is unchanged. The dilution effects, resulting from addition of finite volume of an acid or base to such dynamic systems during titrations, was considered in the papers [2, 10], where finite changes (ΔpH) in pH, affected by addition of the strong acid or base, were closely related to the formulas for the acid–base titration curves. The ΔpH values, called ‘windows’, were considered later [11] for a mixture of monoprotic acids titrated with MOH; the dynamic version of this concept was presented first in [10].

Buffering action is involved with mixing of two (usually aqueous) solutions. The mixing can be performed according to the titrimetric mode. In the present paper, the formula for dynamic buffer capacity, $\beta_{V} = \left| {\frac{{{\text{d}}c}}{\text{dpH}}} \right|$ related to the systems where *V*_0_ mL of the solution being titrated (titrand, D) of different complexity, with concentrations [mol·L^−1^] of component(s) denoted by *C*_0_ or *C*_0*k*_, is titrated with *V* mL of *C* mol·L^−1^ solution of: MOH (e.g. NaOH), HB (e.g. HCl), or a weak polyprotic acid H_*n*_L or its salt of M_*m*_H_*n*−*m*_L (*m* = 1,…,*n*), or H_*n*+*m*_LB_*m*_ type as a reagent in titrant (T) are considered. This way, the D + T mixture of volume *V*_0_ + *V* mL, is obtained, if the assumption of additivity of the volumes is valid. It is assumed that, at any stage of the titration, D + T is a mono-phase system where only acid-base reactions occur. The formation function $\bar{n} = \bar{n}(pH)$ [12, 13] was incorporated, as a very useful concept, into formulas for acid-base titration curves, obtained on the basis of charge and concentration balances, referred to polyprotic acids.

## Definition of Dynamic Buffer Capacity

In this work, the buffer capacity is defined as follows:

1 $$ \beta_{V} = \left| {\frac{{{\text{d}}c}}{\text{dpH}}} \right| $$

where

2 $$ c = C \cdot \frac{V}{{V_{0} + V}} \equiv C - \frac{{C \cdot V_{0} }}{{V_{0} + V}} $$

denotes the current concentration of a reagent R in a D + T mixture obtained after addition of *V* mL of *C* mol·L^−1^ solution of the reagent R (considered as titrant, T) into *V*_0_ mL of a solution named as titrand (D). From Eqs. 1 and 2 we have:

3 $$ \beta_{V} = \left| {\frac{{{\text{d}}c}}{{{\text{d}}V}} \cdot \frac{{{\text{d}}V}}{\text{dpH}}} \right| = \frac{{C \cdot V_{0} }}{{(V_{0} + V)^{2} }} \cdot \left| {\frac{{{\text{d}}V}}{\text{dpH}}} \right| $$

The buffer capacity *β*_*V*_ is an intensive property, expressed in terms of molar concentrations, i.e., intensive variable. The expressions for $\frac{{{\text{d}}V}}{\text{dpH}}$ in Eq. 3 will be formulated below.

## Formulation of Dynamic Buffer Capacity

Some particular systems can be distinguished. For the sake of simplicity in notation, the charges of particular species $X_{i}^{{z_{i} }}$ will can be omitted when put in square brackets, expressing molar concentration $\left[ {X_{i} } \right]$.

**System 1A**: *V* mL of MOH (*C*, mol·L^−1^) is added, as reagent R, into *V*_0_ mL of K_*m*_H_*n*−*m*_L (*C*_0_, mol·L^−1^). The concentration balances are as follows:

4 $$ \left[ {\text{M}} \right] \, = CV/\left( {V_{0} + V} \right); \, \left[ {\text{K}} \right] \, = m \cdot C_{0} V_{0} /\left( {V_{0} + V} \right);\,\,\sum\limits_{i = 0}^{q} {[{\text{H}}_{i} {\text{L}}]} = \frac{{C_{0} V_{0} }}{{V_{0} + V}} $$

Denoting:

$$ [{\text{H}}_{i} {\text{L}}] = K_{i}^{\text{H}} \cdot [{\text{H}}]^{i} \cdot [{\text{L}}];\,\,b_{i} = K_{i}^{\text{H}} \cdot [{\text{H}}]^{i} ;\,\,f_{i} = \frac{{b_{i} }}{{\sum\limits_{j = 0}^{q} {b_{j} } }} $$

5 $$ \alpha = \, \left[ {\text{H}} \right] \, {-} \, \left[ {\text{OH}} \right]\,\, = \,\,10^{{ - {\text{pH}}}} - 10^{{{\text{pH}} - {\text{p}}K_{\text{W}} }} $$

and applying the formula for mean number of protons attached to L^−*n*^ [2]

6 $$ \bar{n} = \frac{{\sum\limits_{i = 1}^{q} {i \cdot [{\text{H}}_{i} {\text{L}}]} }}{{\sum\limits_{i = 0}^{q} {[{\text{H}}_{i} {\text{L}}]} }} = \frac{{\sum\limits_{i = 0}^{q} {i \cdot K_{i}^{\text{H}} \cdot [{\text{H}}]^{i} } }}{{\sum\limits_{j = 0}^{q} {K_{j}^{\text{H}} \cdot [{\text{H}}]^{j} } }} = \frac{{\sum\limits_{i = 0}^{q} {i \cdot b{}_{i}} }}{{\sum\limits_{j = 0}^{q} {b{}_{j}} }} = \sum\limits_{i = 0}^{q} {i \cdot f{}_{i}} = \sum\limits_{i = 1}^{q} {i \cdot f{}_{i}} $$

in the charge balance equation

7 $$ \alpha + \, \left[ {\text{M}} \right] \, + \, \left[ {\text{K}} \right] \, + \,\sum\limits_{i = 0}^{q} {(i - n)[{\text{H}}_{i} {\text{L}}]} = 0 $$

we get, by turns,

8 $$ \alpha + \frac{C \cdot V}{{V_{0} + V}} + m \cdot \frac{{C_{0} \cdot V_{0} }}{{V_{0} + V}} = (n - \bar{n}) \cdot \frac{{C_{0} \cdot V_{0} }}{{V_{0} + V}} $$

9 $$ V = V_{0} \cdot \frac{{(n - m - \bar{n}) \cdot C_{0} - \alpha }}{C + \alpha } $$

10 $$ \begin{aligned} V_{0} + V &= V_{0} \cdot \frac{{(n - m - \bar{n}) \cdot C_{0} + C}}{C + \alpha } \\ &   = ((n - m) \cdot C_{0} + C) \cdot V_{0} \cdot \frac{1}{C + \alpha } - C_{0} \cdot V_{0} \cdot \frac{{\bar{n}}}{C + \alpha } \\ \end{aligned} $$

Differentiating Eq. 10 gives:

11 $$ \frac{{{\text{d}}(V_{0} + V)}}{\text{dpH}} = \frac{{{\text{d}}V}}{\text{dpH}} = - ((n - m)C_{0} + C) \cdot V_{0} \cdot \frac{1}{{(C + \alpha )^{2} }} \cdot \frac{{{\text{d}}\alpha }}{\text{dpH}} - C_{0} \cdot V_{0} \cdot \frac{{\frac{{{\text{d}}\bar{n}}}{\text{dpH}} \cdot (C + \alpha ) - \bar{n} \cdot \frac{{{\text{d}}\alpha }}{\text{dpH}}}}{{(C + \alpha )^{2} }} $$

Applying the relation:

12 $$ \frac{{{\text{d}}z}}{\text{dpH}} = \frac{{{\text{d}}z}}{\text{d[H]}} \cdot \frac{\text{d[H]}}{\text{dpH}} = - \ln 10 \cdot [ {\text{H]}} \cdot \frac{{{\text{d}}z}}{\text{d[H]}} $$

for z = α (Eq. 5) and $\bar{n}$ (Eq. 6), we get [2, 12]:

13 $$ \frac{{{\text{d}}\alpha }}{\text{dpH}} = - \ln 10 \cdot ([{\text{H}}] + [{\text{OH}}]) $$

14 $$ \frac{{{\text{d}}\bar{n}}}{\text{dpH}} = - \ln 10 \cdot \sum\limits_{j > i = 0}^{q} {(j - i)^{2} \cdot f_{i} f_{j} } $$

and then from Eq. 11 we have:

15 $$ \begin{aligned} \frac{{{\text{d}}V}}{\text{dpH}} &= \frac{{V_{0} \cdot \ln 10}}{{(C + \alpha )^{2} }} \cdot \left.\left(\left((n - m) \cdot C_{0} + C - C_{0} \cdot \sum\limits_{i = 1}^{q} {i \cdot f{}_{i}} \right) \cdot ([{\text{H}}] + [{\text{OH}}])\right.\right. \hfill \\ & \quad + \left.\left.C_{0} \cdot (C + \alpha ) \cdot \sum\limits_{j > i = 0}^{q} {(j - i)^{2} \cdot f_{i} f_{j} } \right)\right. \hfill \\ \end{aligned} $$

Note that [H] + [OH] = (*α*^2^ + 4*K*_W_)^1/2^ [12] (see Eq. 5), where *K*_W_ = [H][OH].

**System 1B**: When *V* mL of HB (*C*, mol·L^−1^) is added into *V*_0_ mL of K_*m*_H_*n*−*m*_L (*C*_0_, mol·L^−1^), we have [B] = *CV*/(*V*_0_+*V*). Then *C* is replaced by −*C* in the related formulas, and we have:

16 $$ \begin{aligned} V &= V_{0} \cdot \frac{{(\bar{n} + m - n) \cdot C_{0} + \alpha }}{C - \alpha } \hfill \\  &= V_{0} \cdot \frac{{ - (\bar{n} + m - n) \cdot C_{0} - \alpha }}{ - C + \alpha } = V_{0} \cdot \frac{{(n - m - \bar{n}) \cdot C_{0} - \alpha }}{ - (C - \alpha )} \hfill \\ \end{aligned} $$

As we see, Eq. 16 can be obtained by setting −*C* for *C* in the related formula. Applying it to Eq. 15, we get

17 $$ \begin{aligned} \frac{{{\text{d}}V}}{\text{dpH}} &= \frac{{V_{0} \cdot \ln 10}}{{(C - \alpha )^{2} }} \cdot  \left.\left(\left((n - m) \cdot C_{0} - C - C_{0} \cdot \sum\limits_{i = 1}^{q} {i \cdot f{}_{i}} \right) \cdot ([{\text{H}}] + [{\text{OH}}])\right.\right.  \\ & \quad \left.\left.-\, C_{0} \cdot (C - \alpha ) \cdot \sum\limits_{j > i = 0}^{q} {(j - i)^{2} \cdot f_{i} f_{j} } \right) \right.   \\ \end{aligned} $$

**System 2A**: *V* mL of *C* mol·L^−1^ MOH is added into *V*_0_ mL of the mixture: ${\text{K}}_{{m_{k} }} {\text{H}}_{{n_{k} - m_{k} }} {\text{L}}_{\left( k \right)}$(*C*_0*k*_; *m*_*k*_ = 0,…,*n*_*k*_; *k* = 1,…,P); ${\text{H}}_{{n_{k} + m_{k} }} {\text{L}}_{(k)} {\text{B}}_{{m_{k} }}$(*C*_0*k*_; *m*_*k*_ = 0,…,*q*_*k*_ − *n*_*k*_; *k* = P+1,…,Q), HB (*C*_0a_) and MOH (*C*_0b_). Denoting −*n*_*k*_—charge of ${\text{L}}_{(k)}^{{ - n_{k} }}$, we have the charge balance equation:

18 $$ \alpha + [{\text{K}}] + [{\text{M}}] - [{\text{B}}] + \sum\limits_{k = 1}^{Q} {\sum\limits_{j = 0}^{{q_{k} }} {(j - n_{k} )[{\text{H}}_{j} {\text{L}}_{k} ] = 0} } $$

where:

19 $$ \sum\limits_{j = 0}^{{q_{k} }} {[{\text{H}}_{j} {\text{L}}_{(k)} ]} = \frac{{C_{0k} V_{0} }}{{V_{0} + V}}\,\,\left( {k = { 1}, \ldots ,{\text{ P}},{\text{ P}} + 1, \ldots ,{\text{Q}}} \right);\,\,[{\text{K}}] = \frac{{\sum\limits_{k = 1}^{P} {m_{k} C_{0k} V_{0} } }}{{V_{0} + V}} $$

20 $$ [{\text{M}}] = \frac{{CV + C_{0b} V_{0} }}{{V_{0} + V}} $$

21 $$ [{\text{B}}] = \frac{{\sum\limits_{k = P + 1}^{Q} {m_{k} C_{0k} } V_{0} + C_{0a} V_{0} }}{{V_{0} + V}} $$

The presence of strong acid HB (*C*_0a_) and MOH (*C*_0b_) in the titrand D can be perceived as a kind of pre-assumed/intentional “mess” done in stoichiometric composition of the salts. Denoting: [H_*i*_L_(*k*)_] = *K*_*ki*_^H^·[H]^*i*^·[L_(*k*)_]; *b*_*ki*_ = *K*_*ki*_^H^·[H]^*i*^, and

22 $$ f_{ki} = \frac{{b_{ki} }}{{\sum\limits_{j = 0}^{{q_{k} }} {b_{kj} } }};\,\,\,\bar{n}_{k} = \frac{{\sum\limits_{i = 0}^{{q_{k} }} {i \cdot [{\text{H}}_{i} {\text{L}}_{\left( k \right)} ]} }}{{\sum\limits_{j = 0}^{{q_{k} }} {[{\text{H}}_{j} {\text{L}}_{\left( k \right)} ]} }} = \frac{{\sum\limits_{i = 0}^{{q_{k} }} {iK_{ki}^{\text{H}} \cdot [{\text{H}}]^{i} } }}{{\sum\limits_{j = 0}^{{q_{k} }} {K_{kj}^{\text{H}} \cdot [{\text{H}}]^{j} } }} = \frac{{\sum\limits_{i = 0}^{{q_{k} }} {ib_{ki} } }}{{\sum\limits_{j = 0}^{{q_{k} }} {b_{kj} } }} = \sum\limits_{i = 1}^{{q_{k} }} {i \cdot f_{ki} } $$

we have:

23 $$ \frac{{{\text{d}}\bar{n}_{k} }}{\text{dpH}} = - \ln 10 \cdot \sum\limits_{j > i = 0}^{{q_{k} }} {(j - i)^{2} \cdot f_{ki} f_{kj} } $$

Introducing Eqs. 19–23 into Eq. 18 we get, by turns:

$$ \alpha + \frac{{\sum\limits_{k = 1}^{p} {m_{k} C_{0k} } V_{0} }}{{V_{0} + V}} + \frac{{CV + C_{0b} V_{0} }}{{V_{0} + V}} - \frac{{\sum\limits_{k = p + 1}^{Q} {m_{k} C_{0k} } V_{0} + C_{0a} V_{0} }}{{V_{0} + V}} + \sum\limits_{k = 1}^{Q} {\sum\limits_{i = 0}^{{q_{k} }} {(i - n_{k} )[{\text{H}}_{i} {\text{L}}_{\left( k \right)} ] = 0} } $$

$$ \alpha V_{0} + \alpha V + \sum\limits_{k = 1}^{p} {m_{k} C_{0k} V_{0} + CV + \Delta_{0} V_{0} } - \sum\limits_{k = p + 1}^{Q} {m_{k} C_{0k} V_{0} } - \sum\limits_{k = 1}^{Q} {n_{k} \cdot C_{0k} V_{0} } + \sum\limits_{k = 1}^{Q} {\bar{n}_{k} \cdot C_{0k} V_{0} = 0} $$

24 $$ V_{0} + V = V_{0} \cdot \left(\sum\limits_{k = 1}^{P} {(n_{k} - m_{k} ) \cdot C_{0k} } + \sum\limits_{k = P + 1}^{Q} {(n_{k} + m_{k} ) \cdot C_{0k} } - \Delta_{0} + C\right) \cdot \frac{1}{C + \alpha } - V_{0} \cdot \frac{{\sum\limits_{k = 1}^{Q} {\bar{n}_{k} \cdot C_{0k} } }}{C + \alpha } $$

25 $$ \begin{aligned} \frac{{{\text{d}}V}}{\text{dpH}} &= \frac{{V_{0} \cdot \ln 10}}{{(C + \alpha )^{2} }} \cdot \left. \left(\sum\limits_{k = 1}^{P} {(n_{k} - m_{k} ) \cdot C_{0k} } + \sum\limits_{k = P + 1}^{Q} {(n_{k} + m_{k} ) \cdot C_{0k} } - \sum\limits_{k = 1}^{Q} {C_{0k} \cdot \sum\limits_{i = 1}^{{q_{k} }} {i \cdot f_{ki} - \Delta_{0} + C \Big) \cdot ([H] + [OH])} }\right. \right. \\ & \quad \left. \left.+\, (C + \alpha ) \cdot \sum\limits_{k = 1}^{Q} {C_{0k} \cdot \sum\limits_{j > i = 0}^{{q_{k} }} {(j - i)^{2} \cdot f_{ki} f_{kj} } } \right) \right.  \end{aligned} $$

where

26 $$ \Delta_{0} = C_{0b} - C_{0a} $$

**System 2B**: *V* mL of *C* mol·L^−1^ HB is added into *V*_0_ mL of the mixture: ${\text{K}}_{{m_{k} }} {\text{H}}_{{n_{k} - m_{k} }} {\text{L}}_{\left( k \right)}$ (*C*_0*k*_; *m*_*k*_ = 0,…,*n*_*k*_; *k* = 1,…,P); ${\text{H}}_{{n_{k} + m_{k} }} {\text{L}}_{\left( k \right)} {\text{B}}_{{m_{k} }}$(*C*_0*k*_; *m*_*k*_ = 0,…,*q*_*k*_ − *n*_*k*_; *k* = P+1,…,Q), HB (*C*_0a_) and MOH (*C*_0b_). We have the balances Eqs. 18 and 19, and

27 $$ [{\text{M}}] = \frac{{C_{0b} V_{0} }}{{V_{0} + V}} $$

28 $$ [{\text{B}}] = \frac{{\sum\limits_{k = P + 1}^{Q} {m_{k} C_{0k} } V_{0} + C_{0a} V_{0} + CV}}{{V_{0} + V}} $$

Introducing Eqs. 19, 27, 28 into Eq. 18 and applying Eqs. 13, 22, 23, 26 we obtain:

29 $$ \begin{aligned} \frac{{{\text{d}}V}}{\text{dpH}} &= \frac{{V_{0} \cdot \ln 10}}{{(C - \alpha )^{2} }} \cdot \left.\left(\left.\left(\sum\limits_{k = 1}^{P} {(n_{k} - m_{k} ) \cdot C_{0k} } + \sum\limits_{k = P + 1}^{Q} {(n_{k} + m_{k} ) \cdot C_{0k} } - \,\Delta_{0} - C - \sum\limits_{k = 1}^{Q} {C_{0k} \cdot \sum\limits_{i = 1}^{{q_{k} }} {i \cdot f_{ki} } } \right.\right) \cdot ([{\text{H}}] + [{\text{OH}}]) \right.\right. \\ & \quad \left.\left.- (C - \alpha ) \cdot \sum\limits_{k = 1}^{Q} {C_{0k} \cdot \sum\limits_{j > i = 0}^{{q_{k} }} {(j - i)^{2} \cdot f_{ki} f_{kj} } } \right) \right. \\ \end{aligned} $$

**System 3A**: *V* mL of *C* mol·L^−1^${\text{M}}_{m} {\text{H}}_{n - m} {\text{L}}$ is added into *V*_0_ mL of the mixture: ${\text{K}}_{{m_{k} }} {\text{H}}_{{n_{k} - m_{k} }} {\text{L}}_{\left( k \right)}$(*C*_0*k*_; *m*_*k*_ = 0,…,*n*_*k*_; *k* = 1,…,P); ${\text{H}}_{{n_{k} + m_{k} }} {\text{L}}_{\left( k \right)} {\text{B}}_{{m_{k} }}$(*C*_0*k*_; *m*_*k*_ = 0,…,*q*_*k*_ − *n*_*k*_; *k* = P+1,…,Q), HB (*C*_0a_) and MOH (*C*_0b_). From charge

30 $$ \alpha + [{\text{K}}] + [{\text{M}}] - [{\text{B}}] + \sum\limits_{k = 1}^{Q} {\sum\limits_{j = 0}^{{q_{k} }} {(j - } } n_{k} )[{\text{H}}_{j} {\text{L}}_{\left( k \right)} ] + \sum\limits_{j = 0}^{q} {(j - n)} [{\text{H}}_{j} {\text{L}}] = 0 $$

and concentration balances, Eqs. 19 and 21 and

31 $$ \sum\limits_{j = 0}^{q} {[{\text{H}}_{j} {\text{L}}]} = \frac{CV}{{V_{0} + V}} $$

32 $$ [{\text{M}}] = \frac{{mCV + C_{0b} V_{0} }}{{V_{0} + V}} $$

after introducing Eqs. 19, 21, 31, 32 into Eq. 30 and applying Eqs. 6, 13, 14, 22, 23 and 26, we obtain:

33 $$ \begin{aligned} V_{0} + V = V_{0} \cdot \left( - \sum\limits_{k = 1}^{P} {(n_{k} - m_{k} )C_{0k} } - \sum\limits_{k = P + 1}^{Q} {(n_{k} + m_{k} )C_{0k} } + (n - m) \cdot C + \Delta_{0} \right) \cdot \frac{1}{{(n - m - \bar{n}) \cdot C - \alpha }}   + V_{0} \cdot \frac{{\sum\limits_{k = 1}^{Q} {\bar{n}_{k} \cdot C_{0k} } - \bar{n} \cdot C}}{{(n - m - \bar{n}) \cdot C - \alpha }} \hfill \\ \end{aligned} $$

and then

34 $$ \begin{aligned} \frac{{{\text{d}}V}}{\text{dpH}} &= \frac{{V_{0} \cdot \ln 10}}{{\left(\left(n - m - \sum\limits_{i = 1}^{q} {i \cdot f_{i} } \right) \cdot C - \alpha \right)^{2} }} \cdot \left(\left(\sum\limits_{k = 1}^{P} {(n_{k} - m_{k} ) \cdot C_{0k} } + \sum\limits_{k = P + 1}^{Q} {(n_{k} + m_{k} ) \cdot C_{0k} } \right.\right.\\ & \quad \left.\left.- \sum\limits_{k = 1}^{Q} {C_{0k} \cdot \sum\limits_{i = 1}^{{q_{k} }} {i \cdot f_{ki} } } + \left(\sum\limits_{i = 1}^{q} {i \cdot f_{i} } - n + m \right) \cdot C - \Delta_{0} \right) \cdot \left(C \cdot \sum\limits_{j > i = 0}^{q} {(j - i)^{2} \cdot f_{i} f_{j} } + [{\text{H}}] + [{\text{OH}}]\right)\right. \\ & \quad  \left.- \left(\left(n - m - \sum\limits_{i = 1}^{q} {i \cdot f_{i} } \right) \cdot C - \alpha \right) \cdot \left(\sum\limits_{k = 1}^{Q} {C_{0k} \cdot \sum\limits_{j > i = 0}^{{q_{k} }} {(j - i)^{2} \cdot f_{ki} f_{kj} } } - C \cdot \sum\limits_{j > i = 0}^{q} {(j - i)^{2} \cdot f_{i} f_{j} } \right)\right) \\ \end{aligned} $$

**System 3B**: *V* mL of *C* mol·L^−1^${\text{H}}_{n + m} {\text{LB}}_{m}$ is added into *V*_0_ mL of the mixture: ${\text{K}}_{{m_{k} }} {\text{H}}_{{n_{k} - m_{k} }} {\text{L}}_{(k)}$(*C*_0*k*_; *m*_*k*_ = 0,…,*n*_*k*_; *k* = 1,…,P); ${\text{H}}_{{n_{k} + m_{k} }} {\text{L}}_{\left( k \right)} {\text{B}}_{{m_{k} }}$(*C*_0*k*_; *m*_*k*_ = 0,…,*q*_*k*_ − *n*_*k*_; *k* = P+1,…,Q), HB (*C*_0a_) and MOH (*C*_0b_). Applying Eqs. 19, 27, 31 and

35 $$ [{\text{B}}] = \frac{{\sum\limits_{k = P + 1}^{Q} {m_{k} \cdot C_{0k} } V_{0} + C_{0a} V_{0} + m \cdot CV}}{{V_{0} + V}} $$

in Eq. 30, we obtain:

36 $$ V_{0} + V = V_{0} \cdot \frac{{ - \sum\limits_{k = 1}^{P} {(n_{k} - m_{k} ) \cdot C_{0k} } - \sum\limits_{k = P + 1}^{Q} {(n_{k} + m_{k} ) \cdot C_{0k} } + \sum\limits_{k = 1}^{Q} {\bar{n}_{k} \cdot C_{0k} } + \Delta_{0} + (n + m - \bar{n}) \cdot C}}{{(n + m - \bar{n}) \cdot C - \alpha }} $$

37 $$ \begin{aligned} V_{0} + V &= V_{0} \cdot \left( - \sum\limits_{k = 1}^{P} {(n_{k} - m_{k} ) \cdot C_{0k} } - \sum\limits_{k = P + 1}^{Q} {(n_{k} + m_{k} ) \cdot C_{0k} } + (n + m) \cdot C + \Delta_{0} \right) \cdot \frac{1}{{(n + m - \bar{n}) \cdot C - \alpha }} \hfill \\ & \quad + V_{0} \cdot \frac{{\sum\limits_{k = 1}^{Q} {\bar{n}_{k} \cdot C_{0k} } - \bar{n} \cdot C}}{{(n + m - \bar{n}) \cdot C - \alpha }} \hfill \\ \end{aligned} $$

Then applying Eqs. 6, 13, 14, 23 and 24 in 37, we have:

38 $$ \begin{aligned} \frac{{{\text{d}}V}}{\text{dpH}} &= \frac{{V_{0} \cdot \ln 10}}{{\left(\left(n + m - \sum\limits_{i = 1}^{q} {i \cdot f_{i} } \right) \cdot C - \alpha \right)^{2} }} \cdot \left.\left(\sum\limits_{k = 1}^{P} {(n_{k} - m_{k} ) \cdot C_{0k} }\right.\right. + \sum\limits_{k = P + 1}^{Q} {(n_{k} + m_{k} ) \cdot C_{0k} } \hfill \\ &\quad \left.\left.- (n + m) \cdot C - \Delta_{0} - \sum\limits_{k = 1}^{Q} {C_{0k} \cdot \sum\limits_{i = 1}^{{q_{k} }} {i \cdot f_{ki} } } + C \cdot \sum\limits_{i = 1}^{q} {i \cdot f_{i} } \right)\right. \cdot \left(C \cdot \sum\limits_{j > i = 0}^{q} {(j - i)^{2} \cdot f_{i} f_{j} } + [{\text{H}}] + [{\text{OH}}]\right) \hfill \\ & \quad - \left(\sum\limits_{k = 1}^{Q} {C_{0k} \cdot \sum\limits_{j > i = 0}^{{q_{k} }} {(j - i)^{2} \cdot f_{ki} f_{kj} } } - C \cdot \sum\limits_{j > i = 0}^{q} {(j - i)^{2} \cdot f_{i} f_{j} } \right)\left((n + m - \sum\limits_{i = 1}^{q} {i \cdot f_{i} } ) \cdot C - \alpha \right) \hfill \\ \end{aligned} $$

In all cases it is assumed that *β*_*V*_ ≥ 0; for this purpose, the absolute value (modulus) was introduced in Eq. 1. An analogous assumption was made for the static buffer capacity (*β*).

## Britton–Robinson Buffers (BRB)

**Fig. 1:**
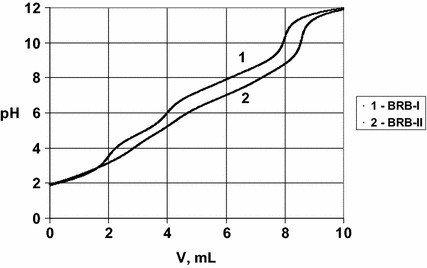
Curves of titration of BRB-I and BRB-II with NaOH. For details see the text

Two buffers proposed by Britton and Robinson [14], marked as BRB-I and BRB-II, are obtained by titration to the desired pH value over the pH range 2–12 [15]. The D (*V* = 10 mL) in BRB-I, consisting of H_3_BO_3_ (*C*_01_) + H_3_PO_4_ (*C*_02_) + CH_3_COOH (*C*_03_), is titrated to the desired pH with NaOH (*C*) as T; in this case, *C*_01_ = *C*_02_ = *C*_03_ = 0.04 mol·L^−1^, and *C* = 0.2 mol·L^−1^. The D in BRB-II, consisting of H_3_BO_3_ (*C*_01_) + KH_2_PO_4_ (*C*_02_) + citric acid H_3_L_(3)_ (*C*_03_) + veronal HL_(4)_ + HCl (*C*_0a_), is titrated to the desired pH with NaOH (C) as T; in this case *C*_01_ = *C*_02_ = *C*_03_ = *C*_04_ = *C*_0a_ = 0.0286 mol·L^−1^, and *C* = 0.2 mol·L^−1^. For BRB-I we have the equation for the titration curve:

39 $$ V = V_{0} \cdot \frac{{(3 - \bar{n}_{1} ) \cdot C_{01} + (3 - \bar{n}_{2} ) \cdot C_{02} + (1 - \bar{n}_{3} ) \cdot C_{03} - \alpha }}{C + \alpha } $$

(see Fig. 1), where:

40 $$ \bar{n}_{1} = \, ( 3\times 10^{{ 3 4. 2 4- 3 {\text{pH}}}} + 2\times 10^{{ 2 5. 7- 2 {\text{pH}}}} + 10^{{ 1 3. 3- {\text{pH}}}} )/( 10^{{ 3 4. 2 4- 3\cdot {\text{pH}}}} + 10^{{ 2 5. 7- 2 {\text{pH}}}} + 10^{{ 1 3. 3- {\text{pH}}}} + 1) $$

41 $$ \bar{n}_{2} = \, ( 3\times 10^{{ 2 1- 7 1 {\text{pH}}}} + 2\times 10^{{ 1 9. 5 9- 2 {\text{pH}}}} + 10^{{ 1 2. 3 8- {\text{pH}}}} )/( 10^{{ 2 1. 7 1- 3 {\text{pH}}}} + 10^{{ 1 9. 5 9- 2 {\text{pH}}}} + 10^{{ 1 2. 3 8- {\text{pH}}}} + 1) $$

42 $$ \bar{n}_{3} = { 1}0^{{ 4. 7 6- {\text{pH}}}} /( 10^{{ 4. 7 6- {\text{pH}}}} + 1) $$

**Fig. 2:**
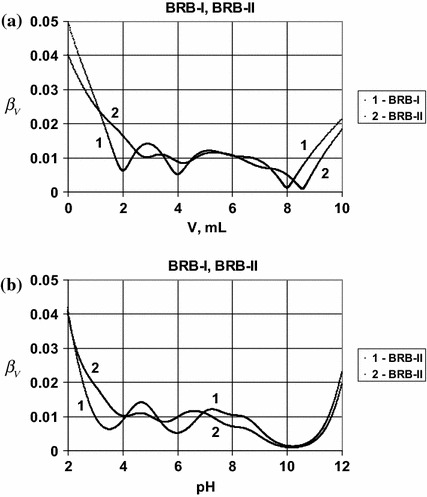
The plots of **a** *β*_*V*_ vs. *V* and **b** *β*_*V*_ vs. pH relationships obtained for BRB-I and BRB-II. For details see the text

For the BRB-II buffer we have the equation for titration curve

43 $$ V = V_{0} \cdot \frac{{(3 - \bar{n}_{1} ) \cdot C_{01} + (2 - \bar{n}_{2} ) \cdot C_{02} + (3 - \bar{n}_{3}^{ \bullet } ) \cdot C_{03} + (1 - \bar{n}_{4} ) \cdot C_{04} + C_{0a} - \alpha }}{C + \alpha } $$

(see Figs. 1, 2), where $\bar{n}_{1}$(Eq. 40) and $\bar{n}_{2}$(Eq. 41) and:

44 $$ \bar{n}_{3}^{ \bullet } = \, ( 3\times 10^{{ 1 4. 2 8- 3 {\text{pH}}}} + 2\times 10^{{ 1 1. 1 5- 2 {\text{pH}}}} + 10^{{ 6. 3 9- {\text{pH}}}} )/( 10^{{ 1 4. 2 8- 3 {\text{pH}}}} + 10^{{ 1 1. 1 5- 2\cdot{\text{pH}}}} + 10^{{ 6. 3 9- {\text{pH}}}} + 1) $$

45 $$ \bar{n}_{4} = { 1}0^{{ 7. 4 3- {\text{pH}}}} /( 10^{{ 7. 4 3- {\text{pH}}}} + 1) $$

The formulas for $\bar{n}_{i}$ (*i* = 1,…,4) and $\bar{n}_{3}^{ \bullet }$ in Eqs. 39 and 43 were obtained on the basis of p*K*_*i*_ values found in [16–20].

Note that

46 $$ \begin{aligned} \sum\limits_{j > i = 0}^{3} {(j - i)^{2} \cdot f_{ki} f_{kj} = f_{k1} f_{k0} + 4f_{k2} f_{k0} + 9f_{k3} f_{k0} + f_{k2} f_{k1} + 4f_{k3} f_{k1} + f_{k3} f_{k2} ;} \hfill \\ \sum\limits_{j > i = 0}^{2} {(j - i)^{2} } \cdot f_{ki} f_{kj} = f_{k1} f_{k0} + 4f_{k2} f_{k0} + f_{k2} f_{k1} ;\,\sum\limits_{j > i = 0}^{1} {(j - i)^{2} } \cdot f_{ki} f_{kj} = f_{k1} f_{k0}. \hfill \\ \end{aligned} $$

## Final Comments

The mathematical formulation of the dynamic buffer capacity *β*_*V*_ concept is presented in a general and elegant form, involving all soluble species formed in the system where only acid–base reactions are involved. This approach to buffer capacity is more general than one presented in the earlier study [2] and is correct from a mathematical viewpoint, in contrast to the one presented in [21]. It is also an extension of an earlier approach, presented for less complex acid–base static [8] and dynamic [10, 12] systems. The calculations were exemplified with two complex buffers, proposed by Britton and Robinson [14].

The salts specified in particular systems considered above do not cover all possible types of the salts, e.g. (NH_4_)_2_HPO_4_ or potassium sodium tartrate (KNaL) are not examples of the salts of ${\text{K}}_{{m_{k} }} {\text{H}}_{{n_{k} - m_{k} }} {\text{L}}_{(k)}$ or ${\text{H}}_{{n_{k} + m_{k} }} {\text{L}}_{(k)} {\text{B}}_{{m_{k} }}$ type. However, in D, (NH_4_)_2_HPO_4_ (*C*_0*i*_) is equivalent to a mixture of NH_3_ (2*C*_0*i*_) and H_3_PO_4_ (*C*_0*i*_), whereas KNaL (*C*_0*j*_) is equivalent to a mixture of NaOH (*C*_0*j*_), KOH (*C*_0*j*_) and H_2_L (*C*_0*j*_).
